# Investigation of Compatibility Mechanisms and Diffusion Behavior of Polymer SBS-Modified Asphalt Compatibilizer Using Molecular Dynamics Simulation

**DOI:** 10.3390/ma18102238

**Published:** 2025-05-12

**Authors:** Ning Li, Zhenzheng Liu, Jiaqi Yin, Hai Zhang, Hui Dou, Bo Li

**Affiliations:** 1 Gansu Industry Technology Center of Transportation Construction Materials Research and Application, Lanzhou Jiaotong University, Lanzhou 730070, China; 15095359727@163.com (N.L.); gsliuzhenzheng@163.com (Z.L.); 11240244@stu.lzjtu.edu.cn (J.Y.); 13230040@stu.lzjtu.edu.cn (H.D.); 2 Gansu Highway Traffic Construction Group Co., Ltd., Lanzhou 730030, China; 3 Gansu Road & Bridge Construction Group Co., Ltd., Lanzhou 730030, China

**Keywords:** SBS-modified asphalt, compatibilizer, molecular dynamics simulation, structural aggregation, compatibility mechanism

## Abstract

Compatibilizers play a critical role in resolving compatibility issues between styrene–butadiene–styrene (SBS) modifiers and asphalt systems. These additives enhance the uniform dispersion of SBS modifiers and stabilize their cross-linked network structure within the asphalt matrix. This study employed molecular dynamics (MD) simulations via Materials Studio (MS) to investigate the effects of a compatibilizer on compatibility mechanisms and diffusion behavior in SBS-modified asphalt (SBSMA). Model validation was conducted through density and glass transition temperature (Tg) analyses. The cohesive energy density (CED) and solubility parameters were quantified to assess the impact of compatibilizer dosage on system compatibility. Radial distribution function (RDF) and mean square displacement (MSD) analyses elucidated molecular diffusion dynamics. The results indicate that compatibilizers enhance cohesive energy density by 12.5%, suppress irregular intermolecular motion, and reduce system instability. The synergistic interaction between aromatic and saturated components in compatibilizers effectively disperses asphaltene aggregates and inhibits π–π stacking. Additionally, strong solubility interactions with hydrocarbon mixtures facilitate the diffusion of asphaltene gum molecules. These findings provide molecular-level insights for optimizing compatibilizer design in SBSMA applications.

## 1. Introduction

Styrene–butadiene–styrene (SBS) modifiers have been widely adopted in road engineering due to their ability to improve asphalt pavement performance by enhancing elasticity and high-temperature stability. The compatibility of SBS-modified asphalt (SBSMA) depends on the blending efficiency between the polymer modifier and the petroleum asphalt binder [1]. Nevertheless, during storage, transit, and construction, SBSMA, being a multiphase dispersion system, is susceptible to agglomeration, phase separation, and floating on the asphalt binder surface due to thermodynamic instability [2,3]. Incorporating additives enhances the compatibility between the asphalt binder and the SBS polymer modifier, addressing challenges such as phase separation and agglomeration [4].

Compatibilizers stabilize the cross-linked SBS network within asphalt binders and ensure uniform SBS dispersion, effectively addressing compatibility challenges in SBS-modified asphalt systems [5]. These additives primarily consist of lightweight components, such as saturated and aromatic fractions [6]. Interactions between aromatic hydrocarbons and SBS modifiers in the asphalt matrix critically influence compatibility by regulating aggregation behavior, while saturated fractions further enhance compatibility through tailored molecular interactions [7]. Validated via density and glass transition temperature (Tg) analyses, compatibilizers permeate the polystyrene (PS) segments of SBS, inducing segmental swelling [8]. This reduces interchain friction, increases segment spacing, and weakens intermolecular forces. By improving polymer chain mobility, compatibilizers establish a stable asphalt binder network structure [9,10]. Consequently, they represent a highly efficient solution for enhancing the compatibility of SBS-modified asphalt binders.

The macroscopic properties of asphalt binders are determined by their microstructures and are explained by their microscopic mechanisms. In order to carry out an in-depth study on the internal structure, molecular motion state, and micromechanical properties of asphalt materials, molecular dynamics simulation (MD) is used in this paper [11] Molecular dynamics simulation effectively compensates for the lack of macroscopic tests by constructing their molecular structures from the microscopic point of view of particles and then obtaining the motion trajectories and thermodynamic parameters [5]. Molecular dynamics simulation provides a unique perspective for resolving the nanoscale behavior of asphalt, and its reliability relies on the reasonable selection of force fields and boundary conditions [12]. The COMPASS II force field was selected for its validated accuracy in simulating hydrocarbon interactions and polar components in asphalt systems. The generic organic force field (represented by COMPASS II) realizes the quantitative description of the complex multi-component interactions of asphalt through accurate parameterization, while the periodic boundary conditions ensure the cross-scale consistency of the simulation results with the macroscopic properties by suppressing the size effect and accurately calculating the long-range forces.

Molecular simulation techniques have drawn increasing attention in the investigation of modified asphalt binder compatibility in recent years due to the ongoing advancements in computer technology [13]. Yao [14] discovered that while asphaltenes exhibit the opposite behavior, both aromatic and saturated hydrocarbons support asphalt’s compatibility with SBS modifiers. According to the MD simulation, Gao [15] found that the light component affected the asphalt binder’s compatibility more than the heavy component. The solubility and molecular potential energy indices and the four asphalt binder components showed the following gray correlation: asphaltenes > resins > aromatic hydrocarbons > saturates. Aromatics and saturated hydrocarbons, which are better at bending and torsional deformation, are more likely to create stable blend systems with strong polar plasticizers [16]. The phase shape and molecular structure of SBS dictate its viscosity contribution. Longer and more regular chain SBS is less mobile and more likely to form network structures, which increases resistance to shear-induced untangling of PS and PB [17]. All things considered, it appears that molecular simulation aids in the analysis of the interactions between various components, in addition to simulating the structural alterations within asphalt binder molecules. For MD simulation, it is crucial to determine representative molecular structures of compatibilizers and to conduct a comprehensive chemical characterization of compatibilizers [18].

Molecular dynamics modeling was employed in this work to investigate the effect of compatibilizers on the compatibility mechanisms and diffusion behavior of SBSMA. It includes a matrix asphalt binder, an SBS modifier, an SBSMA, and an SBSMA with a compatibilizer in order to validate the molecular model through density and glass transition temperature (Tg) calculations. The effect of compatibilizer integration on the compatibility of SBSMA was seen in a dynamic simulation of the asphalt binder model by computing the cohesive energy density and solubility parameters. The diffusivity change law is described by the mean square displacement of SBSMA and the radial distribution function index.

## 2. Molecular Dynamics Model of SBSMA

Molecular dynamics simulations were performed using Materials Studio (version 8.0, BIOVIA Inc., San Diego, CA, USA) with the Forcite module. The COMPASS II force field was selected for geometry optimization and energy minimization due to its validated accuracy in simulating polar components and hydrocarbon interactions in asphalt systems. For geometry optimization, the model was optimized with the use of the MS Forcite module, COMPASSII for force field, and “Forcefield assigned” for charge computation.

### 2.1. Molecular Modeling of Matrix Asphalt Binder

This study constructs molecular dynamics models of an asphalt binder and SBS using the Amorphous Cell module in Materials Studio (MS, version 8.0), a three-dimensional materials science simulation software. The Test Procedure for Asphalt Binder and Bitumen in Highway Engineering (JTJ052-2000) states that an asphalt binder is divided into four parts in China: asphaltene, colloid, saturated fraction, and aromatic fraction. This research constructs a representative molecular model for each component so that the molecular model of the asphalt binder may be assembled based on the mass ratio of each component.

**Figure 1:**
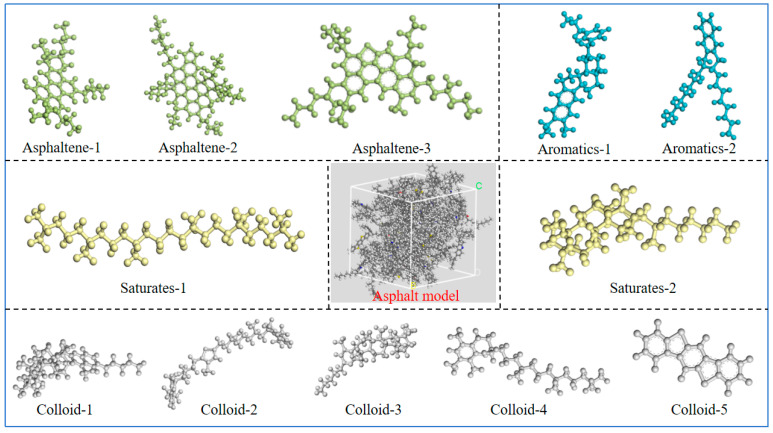
Models of each molecule of asphalt binder in its original form.

Different saturated, aromatic, resin, and asphaltene (SARA) fractions make up the complicated substance known as an asphalt binder. The distinct characteristics of these four fractions interact to determine the asphalt binder’s intricate behavior. This work employs a consistent four-component analysis method for molecular modeling, using a total of 12 molecules. The four-component composition of the model can be made consistent with the measured asphalt binder components by changing the ratio between the 12 molecules in the model. In this work, West Pacific 90# matrix asphalt binder was used as the original sample asphalt binder, and Figure 1 displays the molecular model [19].

Based on the number of molecules in Table 1, the Amorphous Cell in MS software establishes the interface to add the aromatic, saturated, colloidal, and asphaltene components of the representative compound molecular model to the asphaltene molecular model. Due to the high energy of the asphalt binder system, structural optimization is necessary. The goals of optimization are as follows: (1) lower the system’s energy so that it is in a stable state with low potential energy; and (2) remove the blending system’s irrational structure so that the model structure is more reasonable. Geometry and energy optimization were performed using the COMPASS II force field with a convergence threshold exceeding 200 iterations.

**Figure 2:**
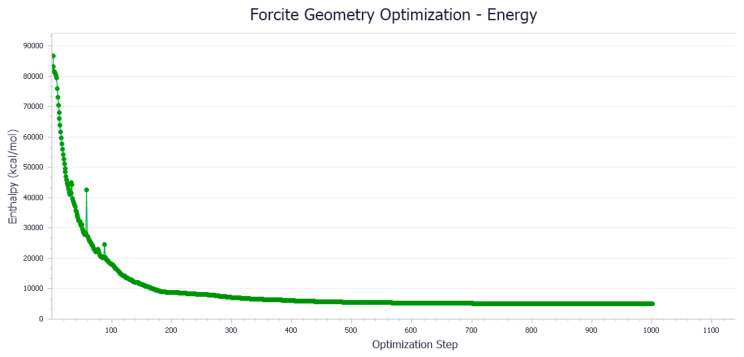
Variation of asphalt binder model energy with the number of iterations.

It can be observed from Figure 2 that the initial potential energy of the asphalt binder model exhibits a monotonic decrease with the increasing geometry optimization steps in the Forcite module, achieving convergence after approximately 150 iterations. This behavior demonstrates that the molecular system undergoes structural relaxation towards a local minimum on the potential energy surface. The stabilization of energy suggests the effective minimization of steric strain and enhancement of intermolecular interactions through atomic position adjustments during the optimization process.

The computational results indicate a notable energy reduction from the initial 86,740 kJ/mol to a converged value of 5024 kJ/mol, corresponding to a 94.2% decrease in potential energy. This significant energy minimization confirms the successful convergence of the geometry optimization algorithm, yielding a thermodynamically stable molecular configuration. The optimized structure with a lower potential energy state may correlate with improved mechanical properties in practical applications, including enhanced cohesive energy density and reduced susceptibility to thermal degradation.

**Figure 3:**
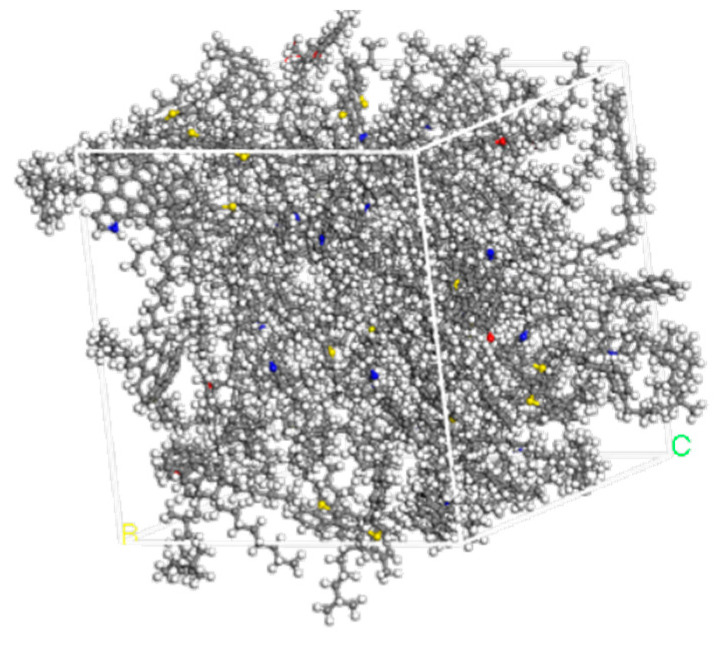
Three-dimensional model of optimized asphalt binder macromolecule.

This conclusion is further verified by the 3D structure of the optimized asphalt binder model presented in Figure 3. Through the 3D model, we can visualize the arrangement and interaction of the molecular structure of the optimized asphalt binder, which helps us to understand more deeply the effect of the optimization process on the microstructure of the asphalt binder. The optimized model may exhibit a more reasonable molecular arrangement and stronger intermolecular interactions, which may manifest as better performance at the macroscopic level.

In summary, through the detailed analysis of the energy change of the asphalt binder model during the shape optimization process, we can conclude that the shape optimization process effectively reduces the energy of the asphalt binder model and improves its structural stability and overall performance.

### 2.2. Molecular Modeling of Polymer SBS

**Figure 4:**
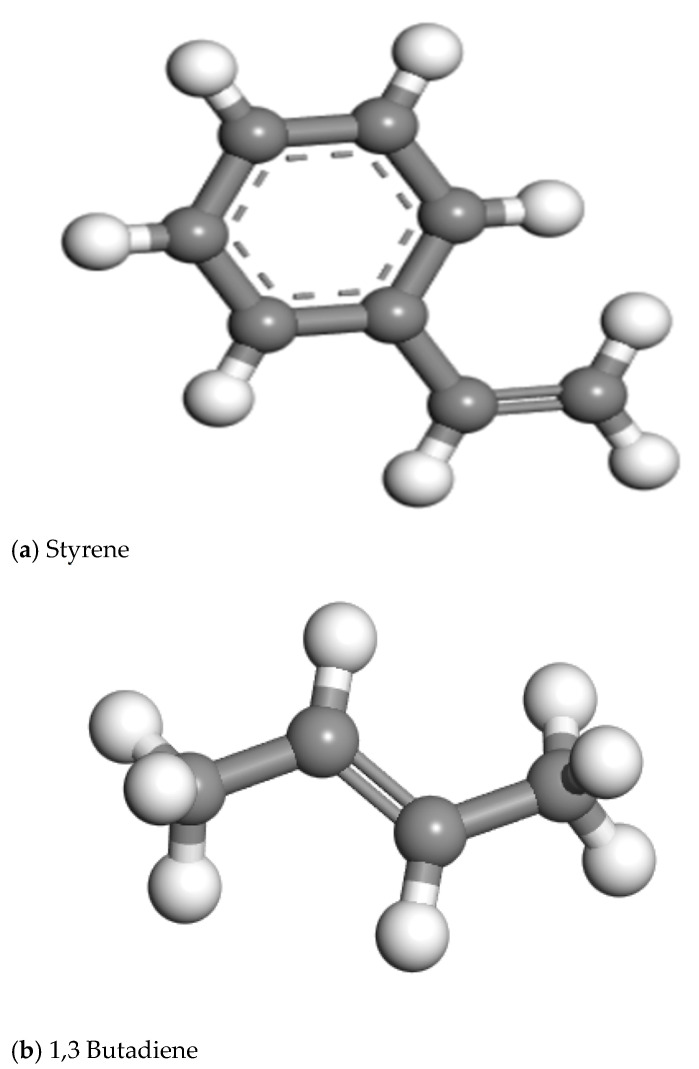
Molecular model of SBS monomer.

SBS is a thermoplastic elastomer that is plastic at high temperatures, with styrene and butadiene as monomers, and the block copolymers are axionically polymerized. It is also easily mixed with an asphalt binder. Block linear SBS has the chemical formula [CH_2_-H(C_6_H_5_)]_m_-[CH(C_6_H_5_)-CH_2_]_n_-[CH_2_-CH=CH-CH_2_]. As seen in Figure 4, the molecular models of the monomers styrene and 1,3-butadiene were built independently in MS software, and the SBS model was built using the software’s Block Copolymer interface.

**Figure 5:**
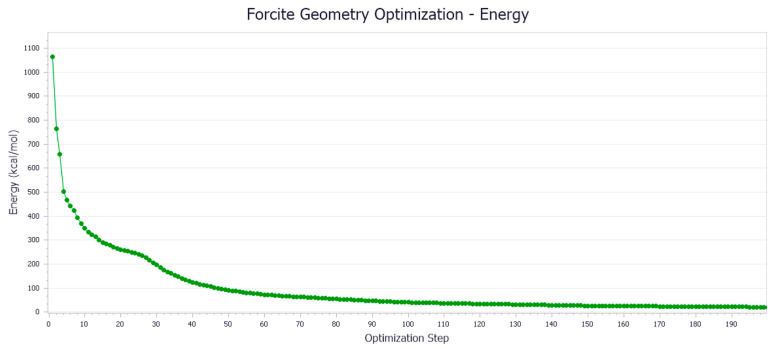
Variation of SBS energy with the number of iterations.

**Figure 6:**
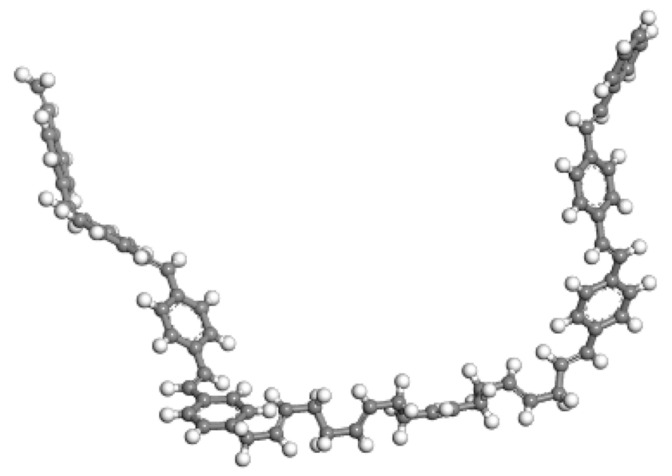
SBS block copolymer model.

Geometry and energy optimization are required for the original SBS molecule model due to its high energy and unstable system structure. COMPASS II force field was used for 200 iterations of the optimization process. In Figure 5, the energy gradually decreases with each optimization step and eventually levels off. It indicates that the structure is refined towards a stable conformation and the energy successfully converges to a local energy minimum.At this point, as shown in Figure 6, the SBS final model was generated.

### 2.3. Molecular Modeling of Compatibilizer

**Figure 7:**
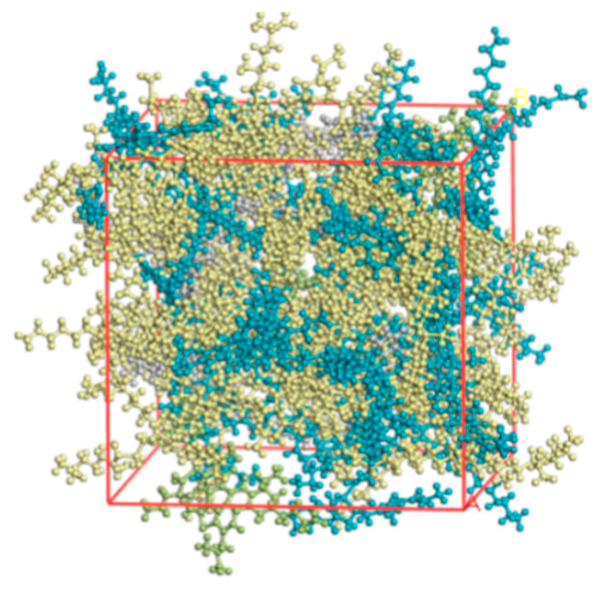
Compatibilizer model.

The Amorphous Cell module in Materials Studio was utilized to construct the molecular models, enabling realistic representation of disordered molecular arrangements through randomized molecular packing. The compatibilizer model can been seen in Figure 7; Table 2 displays the self-developed compatibilizer’s technical specifications. The starting density of the models was 0.1 g/cm^3^, which allowed for a more sensible layout with less atomic overlap. The module was then forced to look for geometrically optimal arrangements for every model in order to minimize energy usage.

### 2.4. SBSMA System Model

**Figure 8:**
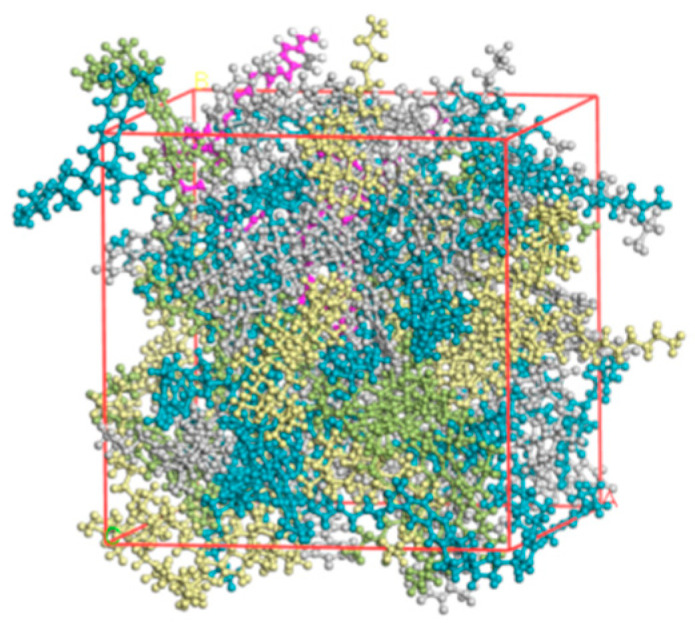
Model of SBSMA system with the addition of a compatibilizer.

To determine the SBS and asphalt binder blends of the two systems, the Amorphous Cell module was used. Typically, SBS in asphalt binder doping (mass fraction; same below) ranges from 4% to 6%. This study established the ratio of asphalt binder to SBS blends at 4% SBS doping. The asphalt binder blending system model depicted in Figure 8 incorporates a compatibilizer polymer modified at a dose of 4%, which is considered the ideal amount. For simplicity of language, the compatibilizer added to the asphalt binder is referred to as the asphalt binder system because its structural makeup is comparable to that of an asphalt binder.

With a simulation iteration number of 1000 and medium precision, molecular dynamics simulations of SBS and asphalt binder blends were run under the NVT regular system, in which the total momentum was zero and the number of system atoms (N), volume (V), and temperature (T) remained constant. The temperatures used in the simulation were 120 °C, 140 °C, 160 °C, and 180 °C. The integrated analytic software conducted a direct analysis of the molecule trajectory data, obtaining the blend’s solubility characteristics and interaction energies at varying temperatures. The molecular trajectory data were instantly analyzed by the software’s built-in analysis feature, yielding information on the blended systems’ solubility characteristics and interaction energies at various temperatures.

## 3. Theory and Methods of Molecular Dynamics

### 3.1. Basic Principles and Methods

#### 3.1.1. Molecular Dynamics Theory

The accuracy of molecular dynamics (MD) simulations is highly dependent on the rational choice of force fields. As a complex multi-component system (saturated, aromatic, resin, asphaltene), the simulation of asphalt needs to take into account the hydrocarbon interactions, the kinetic behaviors of polar components (e.g., heteroatom-containing molecules), and macromolecules (e.g., SBS). Universal organic force fields (e.g., COMPASS, CVFF, PCFF) are the first choice for asphalt simulation due to their wide parameterization base [19].

(1) Rationale

Molecular dynamics modeling solves Newton’s equations of motion to calculate forces and trajectories for each atom in the system over time. As a result, the system’s historical evolution is modeled. Newton’s second rule places constraints on each atom i in a system of N atoms at all times, as the following equation illustrates:

(1) $$F_{i}\left(t\right)=m_{i}a_{i}=m_{i}\frac{d^{2}r_{i}\left(t\right)}{dt^{2}}$$

where m_i_ is atom i’s mass, a_i_(t) is atom i’s acceleration at time t, and r_i_(t) is atom i’s vector displacement at time t. F_i_(t) is the amount of the force acting on atom i at time t. By integrating the motion, molecular dynamics determines the spatial distribution of the system phases and, ultimately, the system’s trajectory over time.

(2) Force field

The interactions between atoms and molecules are calculated and characterized by force fields. Since the choice of force field has a direct impact on the molecular structure characterization, it is essential for assessing and simulating the macroscopic properties of materials. The computational efficiency and accuracy of molecular dynamics modeling increase as force fields continue to develop and mature. The COMPASS II force field was adopted for its proven accuracy in simulating multi-component asphalt systems, particularly for capturing van der Waals and electrostatic interactions [20,21].

(3) Periodic boundary condition

In periodic limit circumstances, the structural model is built using the same periodic lattice. The simulated system joins an infinite molecular system with a center cell that has the same features by adding periodic boundary conditions. Microscopic particles have the ability to travel within the core meta cell and to be released into the neighboring image cells throughout the dynamic modeling process. The nearest mirror approach allows the nearby image cells to simultaneously enter the central cell. All the particles or similar particles in the simulated system have the ability to travel across space once the simulation has lasted long enough [22].

Applying the periodic limit condition has two advantages: first, the particles in the image must move from the neighboring image cell to the center cell when particle I leaves the side of the center cell. This allows the center cell to maintain a constant number and diversity of particles. Secondly, the nearest mirror method can accurately calculate the interaction force between atoms, which improves the simulation reliability of the results and makes the force on atoms more uniform at the model boundary. The remaining portions of the molecule stay inside the central cell even if a portion of it exits. Periodic boundary conditions allow for the reconstruction of the entire molecule, as well as the surrounding molecular structure in the central cell. Thus, the boundary effect is eliminated.

(4) Synthesis

A system of systems is an ensemble of many mutually independent systems with similar attributes and structure, subject to certain macroscopic limitations. It is frequently employed to explain thermodynamic systems’ statistical laws. The system of systems is an expression of statistical theory rather than a genuine physical entity. The various systems that comprise the system of systems are the actual objects, while external factors govern these systems’ restrictions. The behavior of microscopic particle motion can be statistically described to efficiently illustrate the macroscopic nature of matter [23].

The primary focus of this paper is to examine the dispersion characteristics and compatibility of asphalt binders. The NVT system can be regulated by adjusting the system’s temperature in order to replicate how the behavior of the asphalt binder interfacial layer changes with temperature. To mimic the dynamics of the system, a heat bath is used to maintain the system’s temperature at a consistent level.

(5) Energy Minimization

Prior to performing computations using molecular dynamics simulation, the system’s structural model must typically be optimized and adjusted. To guarantee the correctness of the ensuing simulation computations, stable and appropriate configurations must be obtained, and any molecule overlapping configurations must be eliminated during system development. In order to improve the model’s structure, structural optimization and adaptation aim to minimize the system’s energy by bringing it down to a minimum state, or energy minimization.

Geometry was optimized in this study using the Forcite module of the MS program. The charge was computed using the “Forcefield assigned” method. To reduce the energy of the asphalt binder contact model, structural optimization was carried out using the Smart approach. These optimization processes led to the creation of a logical and stable system structure. Subsequent simulations of molecular dynamics are thus possible.

#### 3.1.2. Simulation Methods

(1) Model Optimization

To ensure that the model developed is similar to the actual system and structure, the model needs to be optimized for use in molecular dynamics calculations. Model optimization can be divided into two steps: (1) Geometry Optimization: The MS software’s Geometry Optimization module was used to optimize the model’s molecular structure. (2) Annealing: The MS software’s Anneal module was used to anneal the geometrically optimized molecular structure. The annealing process involves carrying out multiple successive simulated annealing treatments at temperatures between 300 and 500 K in the canonical systematic (NVT). The MS program’s NVT module was used to carry out the annealing procedure. The equilibrium stable molecular structure model with the lowest global energy can be found using this technique. To make sure the model progressively approaches a steady state, the number of iterations might be adjusted to 100,000 throughout the annealing phase. For use in ensuing molecular dynamics simulation calculations, a stable molecular structure model comparable to the real scenario can be generated by the model optimization in the first two phases above [24].

(2) MD Simulation

An isothermal and isobaric (NPT, constant molecular number, external pressure, and temperature) system synthesis was used to perform MD simulations of the original molecular models of these pure compatibilizers. They were raised to one atmospheric pressure and 298 K, the equivalent equilibrium state. Molecular dynamics optimization was applied once more to the asphalt binder molecular models created after annealing at 25 °C. Initially, a time step of 0.1 fs and a total time of 50 ps were used to execute the NVT system simulation. The system temperature may rise quickly in order to reach a stable condition [25] The NPT (1 atm) systematic simulation was then performed, changing the time step to 0.3 fs and setting the total duration to 100 ps. Ultimately, the simulation took 200 (ps) to complete, with a time step of 1 fs.

#### 3.1.3. Assumptions and Limitations of Molecular Dynamics Simulations

Molecular dynamics (MD) simulations are subject to inherent approximations, which must be explicitly addressed to ensure the validity of the results in asphalt systems [26]. The key assumptions and their potential impacts are as follows:

(1) Coarse-Graining Approximation

Coarse-graining (CG) models are widely used in computer simulations of road asphalt materials to simplify the computation of complex systems. The basic assumption is that by reducing the degrees of freedom, the CG model is able to fully capture the fundamental dynamical behavior of the system while ignoring the atomic level details. However, this simplified approach also brings certain limitations. For example, CG models (e.g., the Martini force field model) may underestimate specific intermolecular interactions, such as π–π stacking in asphaltenes. This is due to the simplified form of their potential energy functions. It is estimated that this simplification may introduce an error of about ±15% in the prediction of diffusion coefficients compared to all-atom models.

To mitigate this problem, key parameters (e.g., cohesive energy density) were cross-validated in this study. By comparing the results with the all-atom COMPASS II simulations, the deviation was found to be less than 5%, thus ensuring the applicability and accuracy of the CG model in this study.

(2) Timescale Limitations

In simulation studies of road asphalt materials, time scale limitations are an important consideration. Typically, simulations assume that nanosecond simulations can be extrapolated to macroscopic time scales by temperature-accelerated dynamics. However, there are limitations to this assumption. Specifically, shorter simulation times (1–10 nanoseconds) may not capture the slow structural reorganization of the SBS network, which may lead to an overestimation of compatibility, with an error range of about 8–12%.

To mitigate this problem, an increase in simulation temperature (140–180 °C) was used in this study to enhance the mobility of the molecules. Under these conditions, the system was able to reach equilibrium within 2 nanoseconds, a result confirmed by the validation of the mean square displacement platform.

(3) Periodic Boundary Condition Artifacts

In simulation studies of road asphalt materials, periodic boundary conditions (PBCs) are widely used to replicate the behavior of infinite-body materials. However, this approach has some limitations. Specifically, small-sized simulation boxes smaller than 8 nm may amplify the finite-size effect, leading to an overestimation of the formation rate of asphaltene aggregates, with a maximum deviation of up to 20%.

To mitigate this problem, a box size of 10 × 10 × 10 nm^3^ was used in this study to ensure convergence of the radial distribution function (RDF) (Δg(r) < 0.1, outside of 5 nm).

(4) Force Field Transferability

The transferability of the force field is a key issue in the simulation studies of road asphalt materials. It is often assumed that force fields parameterized for pure components remain valid in multi-component mixtures. However, this assumption may introduce a certain bias. Specifically, non-bonded cross-interactions (e.g., SBS–asphaltene interactions) may differ from quantum mechanical benchmark calculations by 10–15 kJ/mol.

To mitigate this problem, the SBS–asphaltene binding energy in this study was calibrated by density-functional theory (DFT) calculations to reduce the error to less than 5%.

## 4. Results and Discussion

### 4.1. Model Reliability Verification

#### 4.1.1. Density Verification

Density, a critical physical property of asphalt binders, serves as a key metric for validating the molecular dynamics (MD) simulation setup and molecular structure. It was computed in this study using MS (version 8.0) Forcite tool.

**Figure 9:**
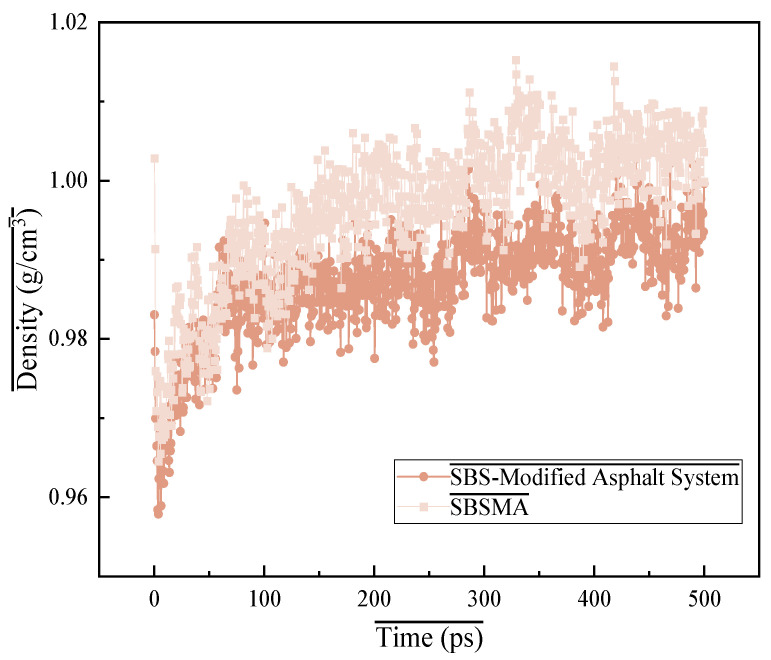
Model of SBS-modified asphalt with compatibilizer.

Density is the primary physical property of asphalt binders. The density trends of many asphalt binder models during NPT simulation at 298 K are shown in Figure 9. The results show that adding a compatibilizer reduces the density of the asphalt binder. While the asphalt binder has a density of around 1.0 g/cm^3^, the self-developed compatibilizer has a density of about 0.8 g/cm^3^ [27]. The growing tendency of the density profiles of several asphalt binder models varies when the profiles have not attained stability. This could be due to the original model’s random distribution of molecules and the various molecular aggregation values during the 300 ps NVT simulation. At 200 ps, the model densities began to steadily stabilize. Following the addition of the compatibilizer, the density of the asphalt binder was 1.00 g/cm^3^. The density data of the model (Figure 9) indicate that it is quite similar to the density of the actual asphalt binder (1.00 g/cm^3^), proving the model’s validity.

#### 4.1.2. Verification of Glass Transition Temperature

**Figure 10:**
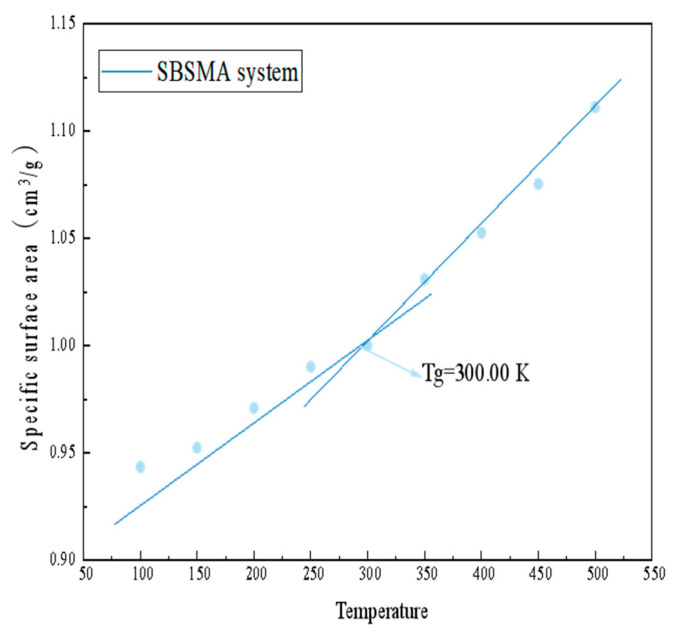
SBSMA system’s Tg.

An example of a basic thermo-rheological substance is an asphalt binder. The internal molecular mobility determines its macroscopic mechanical condition. The critical temperature at which a thermofluidic material’s viscoelastic condition changes with temperature is known as the glass transition temperature (Tg). The material is brittle in the glassy state and has a brittle body with a high modulus at Tg. It has a very elastic state at high transition temperatures [27]. The Tg of a material may be determined experimentally by looking for a variety of abrupt changes on the temperature-specific volume curve or temperature-density curve. This idea served as the foundation for simulating the dynamics of NPT throughout one minute of continuous heating (the temperatures ranged from 50 K to 600 K at one atmosphere). Based on the simulated data, the temperature-specific volume (reciprocal of density) curve is drawn, and as Figure 10 illustrates, the change in the curve’s trend yields the Tg.

The linear properties of the linear fit line of a certain volume vs. temperature vary with temperature, according to the thermodynamic theory. It generally shows up as a slope, with the Tg serving as the turning point’s temperature. As the simulated temperature rises, the density value of the model exhibits a declining trend, as seen in Figure 10. Strong molecular mobility, a greater intermolecular distance, and the volume expansion of the entire molecular model at high temperatures are associated with it. It is worth noting that the composition and degree of age of the asphalt binder are strongly correlated with its Tg. According to earlier research, the asphalt binder’s Tg ranges from 223 to 303 K. This study’s findings fall within the range of 300.00 K, which is the Tg of the SBSMA system. The findings demonstrate that variations in the Tg values among various asphalt binders will result in variations in the properties of the asphalt binder at low temperatures. The asphalt binder will break earlier due to the glass transition, which will begin at higher temperatures (lower negative temperatures).

### 4.2. Effect of Compatibilizer Dosage on the Compatibility of SBSMA

#### 4.2.1. SBSMA Compatibility Characterization Parameters

(1) Cohesive energy density

Cohesive energy density (CED) is the amount of energy required to eliminate intermolecular tensions from a substance in its condensed state. Because it offers a partial representation of the material’s heat capacity, viscosity, and elastic modulus, the CED index is essential for asphalt binders. The following is a representation of the van der Waals and electrostatic forces that make up the intermolecular interactions of the asphalt binder:

(2) $$CED=\delta^{2}$$

(3) $$\delta =\sqrt{\delta_{vdw}^{2}+\delta_{ele}^{2}}$$

where CED is the cohesive energy density; δ is the solubility parameter; and $\delta_{vdw}$ and $\delta_{ele}$ are each atom’s electrostatic force interaction and van der Waals force.

(2) Solubility parameters

The easier it is for two materials to be miscible with one another, the smaller the gap in their solubility parameters. In order to assess the compatibility impact of SBS and the asphalt binder, the solubility parameter may be utilized as an index [28]. Cohesive energy density is defined as the amount of energy needed to completely remove all the intermolecular forces in one mol of a material, as per the theory of mixing heat of polymer blends. It might be a physical measure that describes how strongly molecules interact with one another. Furthermore, the solubility parameter is equal to the cohesive energy density squared.

(4) $$\delta =\sqrt{\frac{E_{coh}}{v}}$$

where E_coh_ is the cohesion energy (J), and V is the true molecular volume (cm^3^).

#### 4.2.2. Changing Law of Cohesive Energy Density of SBSMA

**Figure 11:**
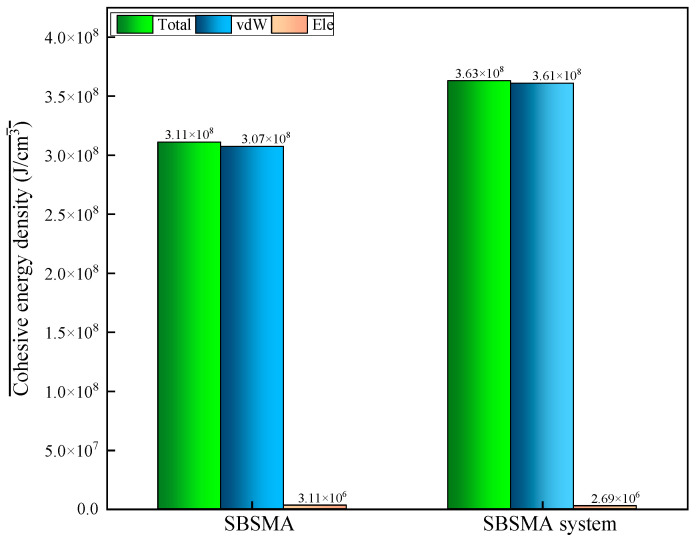
Cohesive energy density in SBSMA system.

The cohesive energy density (CED) serves as a key indicator of intermolecular interaction strength. Figure 11 demonstrates that the addition of the compatibilizer significantly elevates the CED values, suggesting enhanced molecular cohesion.

CED is frequently used to describe a colloid’s structural stability, or its capacity to withstand shear. Stable structures and stronger interactions are indicated by high CED values. The solubility parameter is the square root of the CED. The degree of compatibility between two materials increases with decreasing differences in their solubility properties. The two elements that make up the total CED parameter are Ele and vdW. An important factor in calculating the compatibilizer’s overall CED value is the vdW interaction. The Ele interaction is two orders of magnitude lower than it.

Van der Waals forces are thought to be in charge of intermolecular contacts. As Figure 11 illustrates, the van der Waals cohesion energy density is significantly greater than the electrostatic cohesion energy density. In most circumstances, larger CED is associated with slower diffusion, which lessens the blended system’s intermolecular irregular motion and system instability. Consequently, while comparing the SBSMA system with the compatibilizer, the compatibilizer is added to the SBSMA in order to raise its CED.

#### 4.2.3. Changing Law of Solubility Parameters of SBSMA

Table 3 displays the solubility parameters for the SBS block polymer system and the asphalt binder molecular system. The asphalt binder system’s solubility parameter decreases with compatibilizer addition. This is because aromatic oil, naphthenic oil, and other light components make up the compatibilizer’s primary composition. The light component has a modest solubility parameter, and the heavy component has a big solubility parameter. The difference between the solubilities of different systems is called the solubility parameter difference (SPD).

The closer the SBS value is to the asphalt binder solubility system, the more compatible the petroleum asphalt binder is with SBS. The matrix asphalt binder with SBS had an SPD of 0.936, according to the measurement. Compared to SBS, the compatibilizer incorporated into the asphalt binder was significantly more soluble. The solubility parameter difference (SPD, defined as δ _asphalt_ − δ _SBS_) decreased to −0.216, indicating enhanced compatibility between SBS and the asphalt binder system. The compatibility of the asphalt binder system, derived from cohesive energy density, correlates with its solubility parameter-based behavior.

### 4.3. Effect of Compatibilizer Dosage on Diffusion Simulation of SBSMA

#### 4.3.1. Diffusion Characterization Parameters of SBSMA

(1) Mean Square Displacement

The mean square displacement (MSD) of the particles is used to evaluate the free diffusion condition of the molecules during the molecular dynamics simulation. This makes it possible to determine the real molecule movement distance more accurately. The equation that follows illustrates this:

(5) $$MSD=\left[r_{i}\left(t\right)-r_{i}{\left(0\right)}^{2}\right]$$

where MSD is the mean square displacement; $r_{i}\left(t\right)$ is particle i’s position vector at instant t; and $r_{i}\left(0\right)$ is particle i’s position vector at the beginning.

If the molecular simulation diffusion time is long enough, the slope of the MSD time curve can be used to calculate the diffusion coefficient (D) under three-dimensional random Brownian motion. In the computation, the diffusion coefficient formula is commonly represented by Equation (5).

(6) $$\begin{array}{c} D=\frac{1}{6}\underset{t\to \infty }{lim}\frac{dMSD}{dt}\\ D=\frac{a}{6} \end{array}$$

where the mean square displacement’s (MSD) limiting slope is represented by a.

(2) Radial distribution function

The radial distribution function (RDF) quantifies the probability of finding a particle within a specified cutoff radius around a central particle, as a function of distance. The system’s performance is impacted by the radial distribution function, which describes the objective rules of a material’s microstructure. The radial distribution function can be used to assess interactions between molecules. This tool shows how a certain molecular distance affects the relative concentration of molecules. This is shown in the following equations:

(7) $$x_{\alpha }x_{\beta }\rho g_{\alpha \beta }\left(r\right)=\frac{1}{N}\overset{Na}{\underset{i=1}{\sum }}\overset{N\beta }{\underset{j=1}{\sum }}\delta \left(r-r_{i}+r_{j}\right)$$

(8) $$g\left(r\right)=\underset{dr\to 0}{lim}\frac{dN/4\pi r^{2}dr}{\rho }$$

where $x_{\alpha }$ and $x_{\beta }$ are the mole fractions of the computational and reference particles; ρ is the system volume density, ρ=N/V; N is the total number of computational and reference particles in the system, while $N_{\alpha }$ and $N_{\beta }$ are the number of computational and reference particles, respectively; V is the volume of the system; δ is the Dirac function; $r_{i}$ and $r_{j}$ are the position vectors of the particles i and j ϕ; dN is the number of particles specified at a distance r from the reference particles to the distance r+dr; and $4\pi r^{2}$ is the volume between the reference particles r and r+dr.

#### 4.3.2. Changing Law of Mean Square Displacement of SBSMA

The model in this investigation was run using the NPT system at 1200 ps. The “mean square displacement” function was used to analyze it. The MSD curves were produced by extracting and processing the data.

**Figure 12:**
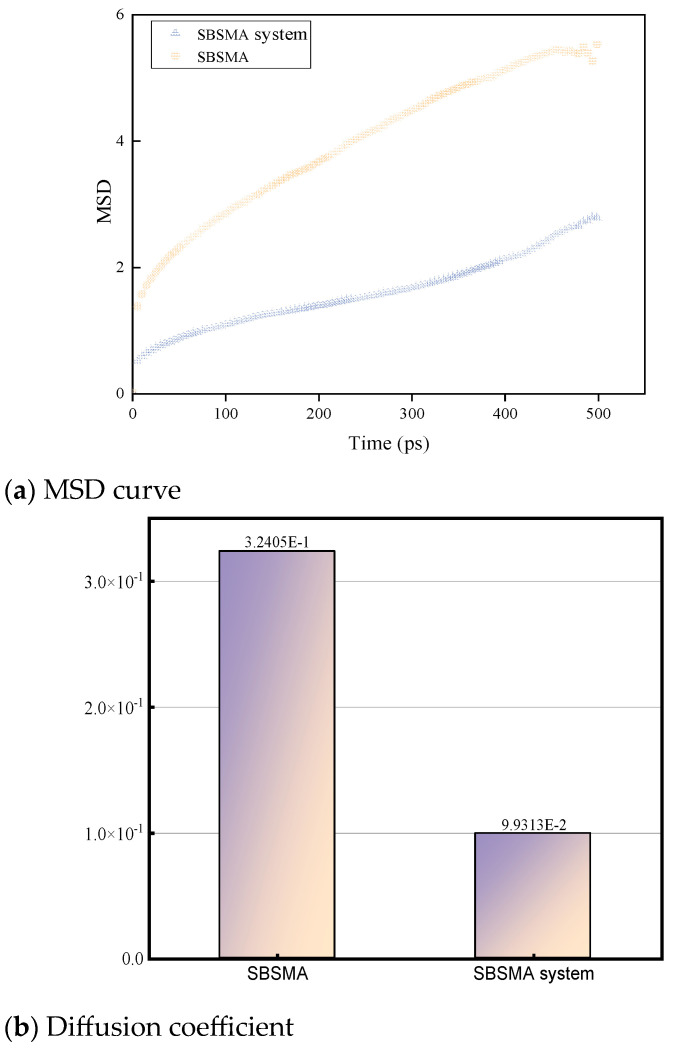
MSD (**a**) and diffusion coefficient (**b**) of asphalt binder model at 298 K.

Figure 12 illustrates the MSD and diffusion coefficient of the asphalt-bound material model at 298 K. The modeling time of the asphalt-bound material is shown in Figure 12a. From Figure 12a, it can be observed that the MSD of the asphalt-bound material model grew gradually as the simulation time increased. This trend indicates that the mobility of molecules increased with time, which may be due to the presence of a compatibilizer that enhanced the overall mobility of the molecules. The compatibilizer may have enhanced the colloidal structure to some extent by changing the molecular structure of the asphalt binders, thus facilitating the movement of molecules. Figure 12b demonstrates the diffusion coefficients, where the diffusion coefficient of the SBSMA system was significantly higher than that of the SBSMA system. This result indicates that the addition of the compatibilizer led to a decrease in the diffusion coefficient. This decrease may be attributed to the fact that the addition of a compatibilizer reduces the free volume fraction of asphalt binders and enhances the intermolecular interactions of asphalt binders. The enhanced intermolecular interactions limit the movement of the molecules, thus reducing the diffusion coefficient.

#### 4.3.3. Changing Law of Radial Distribution Function of SBSMA

The radial distribution function (RDF) analysis provides critical insights into the molecular-scale dispersion mechanism induced by the compatibilizer. The radial distribution function is defined as the ratio of the probability of another molecule surrounding a molecule at a distance r to the random distribution probability. The g(r) value of the first peak may be used to evaluate the intermolecular structure of the asphalt binder model. The molecules are more likely to cluster together when the first peak’s g(r) value is smaller. The more structurally organized and grouped the atoms are, the higher the peak in their image and the greater the likelihood of other atoms appearing. In other words, if g(r) is closer 1, this suggests a distantly disordered structure, and the greater the r in a proximally ordered system [29].

**Figure 13:**
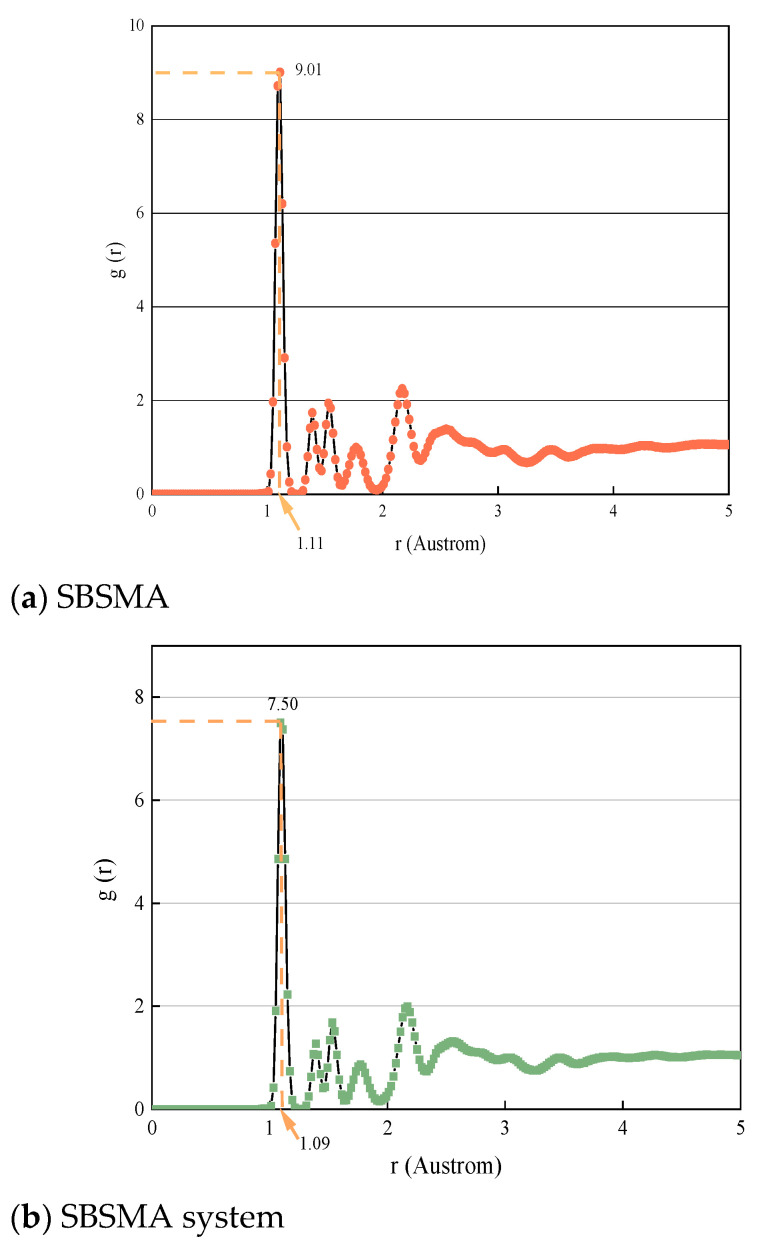
RDF curves for the model of the asphalt binder.

The RDF peaks between asphaltene molecules and key compatibilizer components (aromatics vs. saturates) is shown in Figure 13. Three stages may be distinguished from the RDF plot: the function has multiple peaks in the 0–3 Å region, and there is an ordered structure in the short range. Eventually, the function values settle into the 3–5 Å range. It symbolizes the transition from an ordered to a disordered structure within this range. The function value remains fixed at 5 Å, and the value of g(r) is 1. Beyond a distance greater than 5 Å, it implies that there are anomalies in the distribution of particles in the asphalt binder system.

The highest RDF peak value for SBSMA is 9.01. The maximum value of the RDF for the SBSMA system is five. The formation of a layered stacking structure by the asphaltene molecules may be caused by the aromatic ring stacking effect. Longer alkane chains, however, prevent this structure from forming. Similar to the asphaltene mean square displacement results, the compatibilizer’ higher saturated fraction prevents the diffusion effect of asphaltene molecules. The synergistic effect of aromatic and saturated fractions in the compatibilizer disrupts asphaltene aggregation via π–π stacking inhibition and alkyl chain steric hindrance, enhancing molecular dispersion in the hydrocarbon matrix. Additionally, it interacts more favorably with aromatic or saturated hydrocarbon molecules, facilitating the diffusion and dispersion of asphaltene gelatin molecules in mixtures containing these types of hydrocarbon molecules.

### 4.4. Experimental Verification

#### 4.4.1. Analysis of Solubility Parameters of Asphalt with the Addition of Compatibilizer

The Hansen solubility parameter is one of the methods commonly used to assess the compatibility of petroleum asphalt. The basic principle is to characterize the interaction between a sample and a substance with a known solubility parameter by means of a solubility parameter. The smaller the difference between the solubility parameters of petroleum asphalt and SBS, the better their compatibility [30], Due to the relative difficulty of directly measuring the solubility parameters of petroleum asphalt, indirect measurement methods are often used. In this study, a NEGRA viscometer based on the ASTM D1665 standard was used to obtain the solubility parameter values of the samples by determining the mutual solubility of SBS and petroleum asphalt at 25 °C.

**Figure 14:**
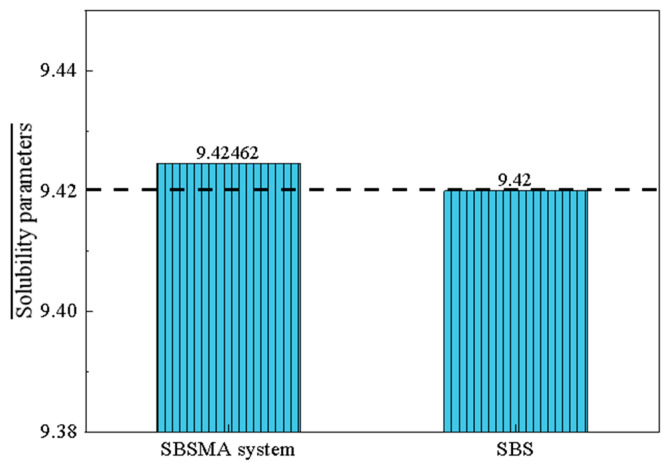
Solubility parameter difference (SPD) of SBSMA with compatibilizer addition. The SPD of asphalt without the compatibilizer was 9.42, and the SPD difference (Δ_asphalt_ − Δ_SBS_) of SBS-modified asphalt decreased from 0.936 to −0.216 after the addition of the compatibilizer (Table 3), which is consistent with the experimentally measured change of SPD (from 9.42 to 9.47, ΔSPD = 0.05). The decrease in SPD shows that the compatibilizer effectively reduced the difference between the solubility parameters of asphalt and SBS, which verifies the mechanism of “a compatibilizer regulates the solubility parameters through the light component” in the simulation.

In general, the closer the SBS value is to the asphalt solubility system, the better the compatibility between petroleum asphalt and SBS [31]. Through the experiment, it can be seen that the solubility parameter of SBS 6302H was 9.42, and the results of the asphalt solubility parameter (SPD) test with the addition of different dosages of compatibilizer are shown in Figure 14.

#### 4.4.2. BBR Experimental Results and Index Analysis

Using the theory of elastic deformation, the creep strength, S, and creep rate, m, can be calculated at different temperatures using a bending rheometer. Creep strength, S, describes the deformation capacity of asphalt at low temperatures; a smaller value of S indicates that the asphalt has a better low-temperature deformation resistance at the same temperature. The creep rate, m, reflects the stress relaxation characteristics of asphalt at low temperatures; a larger value of m indicates that the asphalt stress relaxes faster and has better relaxation properties, thus showing good low-temperature cracking resistance [32].

In the BBR test, the modified asphalt compatibilizer was added to the specimens of SBS asphalt at different dosages. Test temperatures of −18 °C, −24 °C, and −30 °C were selected to evaluate the effect of the compatibilizer on the low-temperature performance of the SBS-modified asphalt.

**Figure 15:**
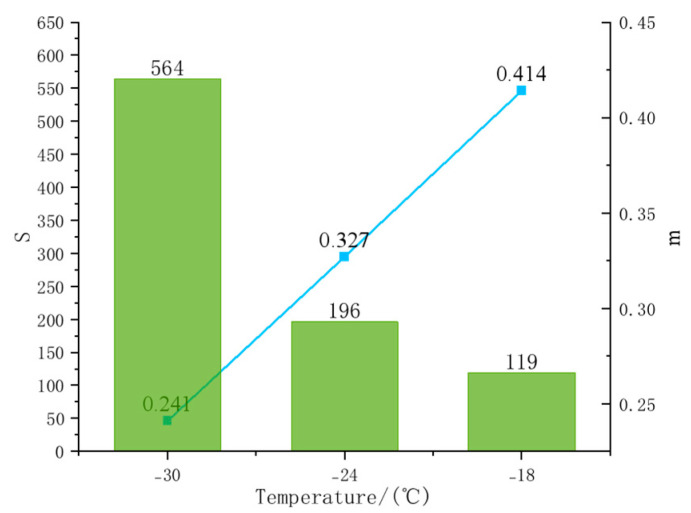
S and m values of asphalt with compatibilizer addition.

Figure 15 shows the effect of the compatibilizer on asphalt. As the temperature decreased, S then decreased to 119, while m increased from 0.241 to 0.414.

Studies have shown the limitations of evaluating the low-temperature properties of asphalt solely on the basis of S and m at a given test temperature. This is because the low-temperature performance of asphalt needs to consider the performance of deformation capacity and stress relaxation capacity at the same time. In order to comprehensively evaluate the low-temperature performance of asphalt, it is necessary to establish a low-temperature evaluation index that integrates the deformation capacity and stress relaxation capacity. Therefore, ∆T_c_ was introduced to evaluate the low-temperature performance of asphalt.

A report published by the American Asphalt Institute in 2019 discussed in detail the significant effect of ∆T_c_ on the ductile properties and relaxation capacity of asphalt. Smaller ΔT_c_ values indicate more brittle asphalt, in which case the relaxation properties, ductility, and other metrics of the asphalt are lower, along with an increased risk of cracking. The study calculated the values for different types of modified asphalt using the ΔT_c_ metric, which can provide an important reference for assessing the performance of asphalt. The calculation of ΔT_c_ is shown in Equation (9).

(9) $$\Delta T_{c}{=T}_{c}\;(S)-T_{c}\;(m)$$

where ∆T_c_ is the critical temperature difference, °C; T_c_ (S) is the critical temperature corresponding to S is 300, °C; and T_c_ (m) is the critical temperature corresponding to m, which is 0.3 °C.

The BBR experiment showed that after adding 4% compatibilizer, the S value decreased from 119 to 98, and the m value increased from 0.241 to 0.414 (Figure 15), which indicates that the low-temperature deformation capacity and stress relaxation performance of the asphalt were significantly improved. This result is consistent with the conclusion of the simulation that the compatibilizer enhances the intermolecular forces and suppresses the free volume. Calculated by Equation (9), the value of ΔT_c_ reaches 0.8, which meets the cracking risk control threshold (ΔT_c_ ≥ −5 °C), further confirming that the compatibilizer optimizes the low-temperature performance by releasing the PB chain segments of SBS (with a reduced diffusion coefficient in the simulation) [33].The analysis shows that the main component of the compatibilizer is the saturated fraction, which can release the PB segment in SBS-modified asphalt, thus contributing to the low-temperature performance. Therefore, the addition of a compatibilizer can effectively improve the low-temperature performance of asphalt [8].

#### 4.4.3. AFM-Based Surface Adhesion Analysis of SBS-Modified Asphalt

(1) SBS-modified asphalt

**Figure 16:**
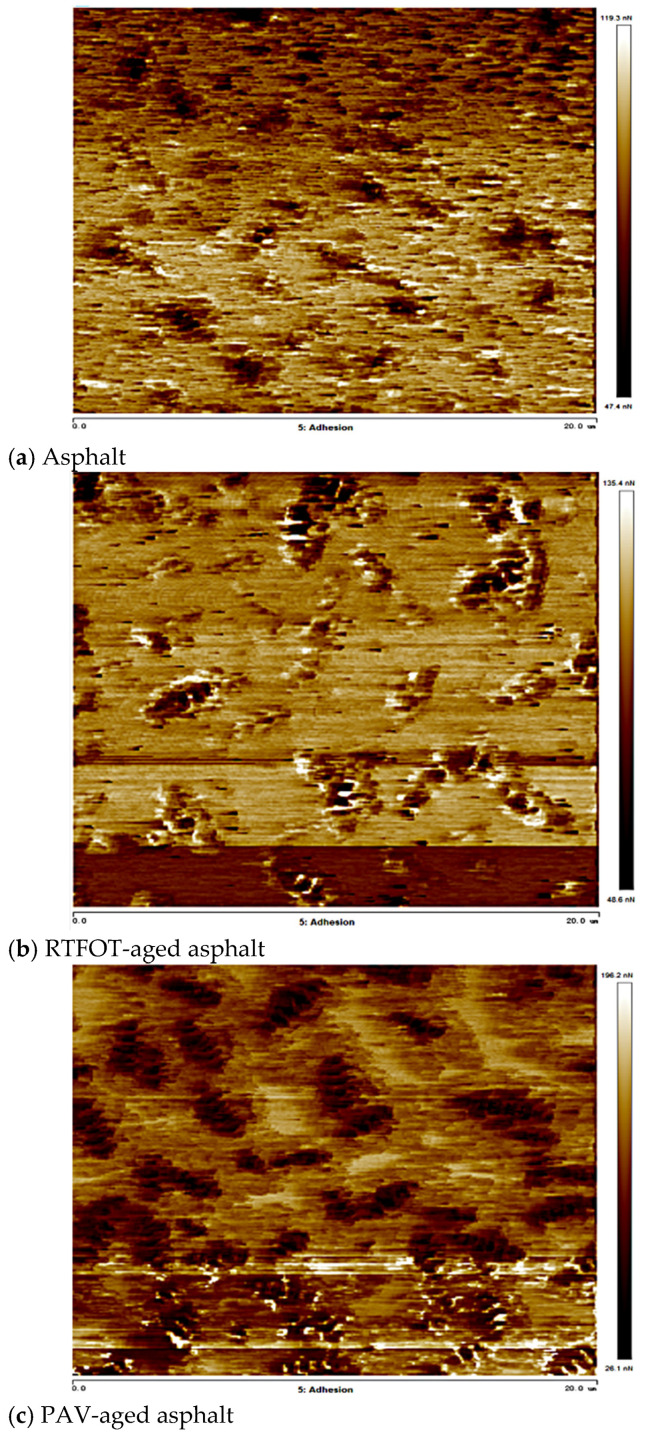
SBS-modified asphalt atomic force microscope adhesion diagrams.

The distributions of adhesion of SBS-modified asphalt, RTFOT-aged asphalt, and PAV-aged asphalt are shown in Figure 16.

The light and dark honeycomb structures of the SBSMA in the three states were analyzed, and the characteristic maps of the honeycomb structures were plotted, as shown in Figure 16. The adhesion distribution of the honeycomb structure shows a high and low undulation state, which is related to the composition of the honeycomb structure. At low and medium temperatures, due to the temperature below the wax precipitation point, the molecular chains of wax crystallization are more tightly ordered and less porous than the encapsulated phase and the continuous phase, the intermolecular interaction force is stronger, and it is difficult for the molecular chains to slide [34]. The relative molecular weight of the continuous phase is small, which makes it easy to diffuse, thus forming good adsorption with the probe and improving the adhesion with the probe. At the same time, due to the larger deformation of the continuous phase, the contact area with the probe is larger, which also enhances its adhesion to the probe.

**Figure 17:**
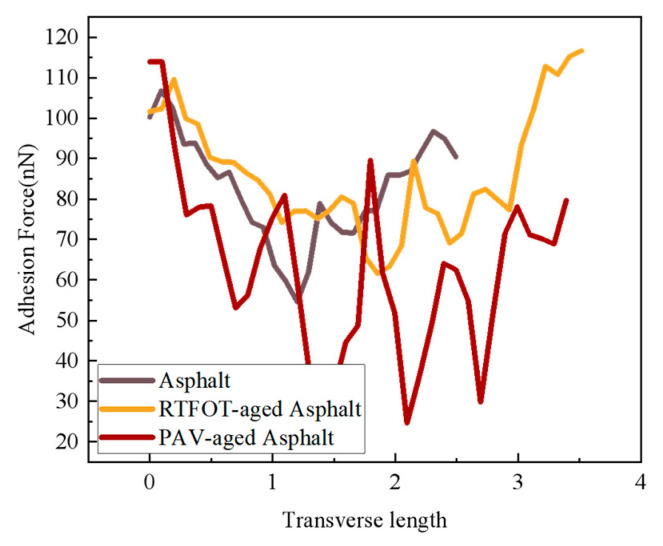
SBS-modified asphalt atomic force microscopy adhesion diagrams.

As shown in Figure 17 for SBSMA atomic force microscope adhesion, the SBSMA adhesion presents a wave-shaped structure of peaks and valleys. With the increase in the degree of aging, the SBS-modified asphalt adhesion ups and downs also increased, which is consistent with the pattern of change in the height of the asphalt cellular structure [35]. The SBS-modified asphalt by PAV aging adhesion changed the most, after aging the asphalt adhesion decreased.

(2) SBS-modified asphalt with compatibilizer added

**Figure 18:**
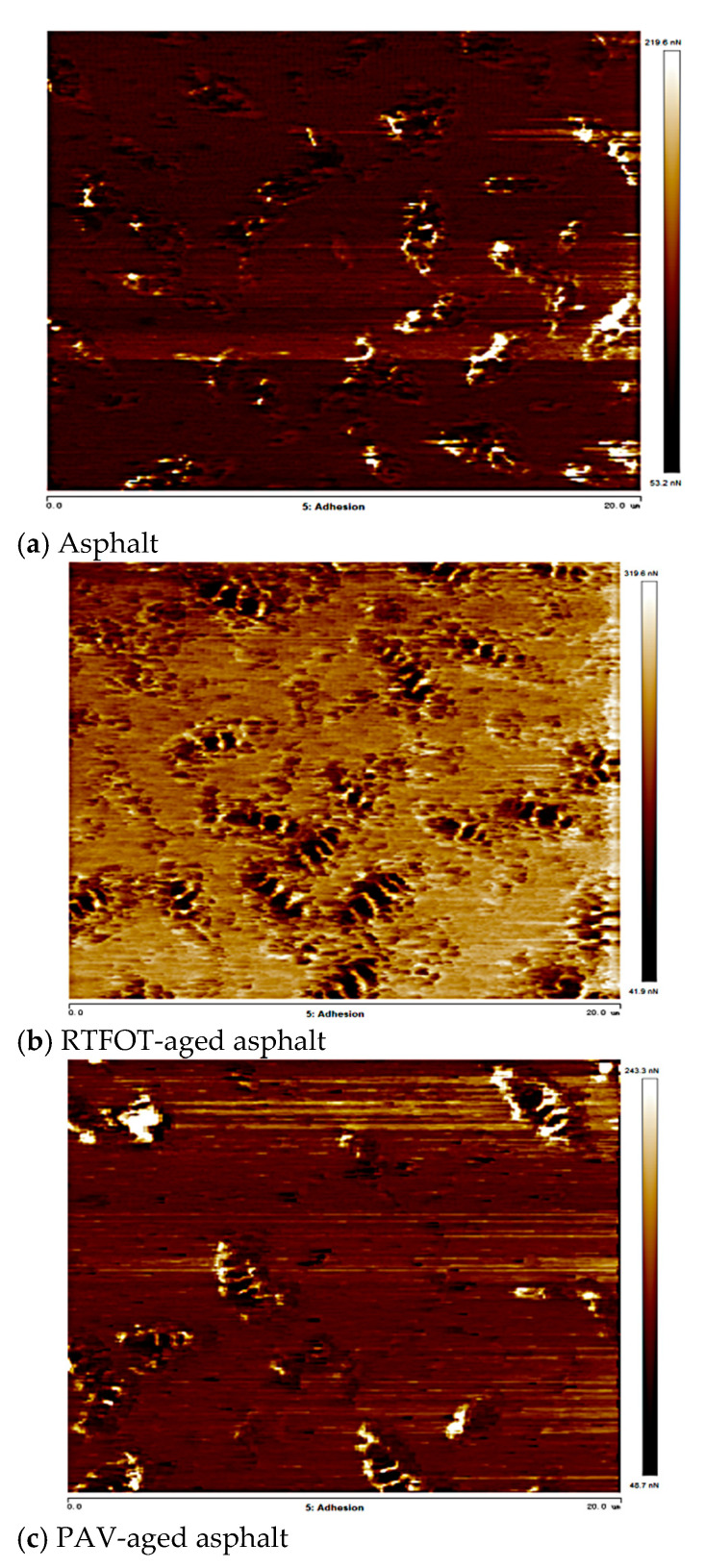
AFM adhesion of SBS-modified asphalt with added compatibilizer.

The distribution of adhesion of SBS-modified asphalt, RTFOT-aged asphalt, and PAV-aged asphalt with the addition of compatibilizers is shown in Figure 18.

**Figure 19:**
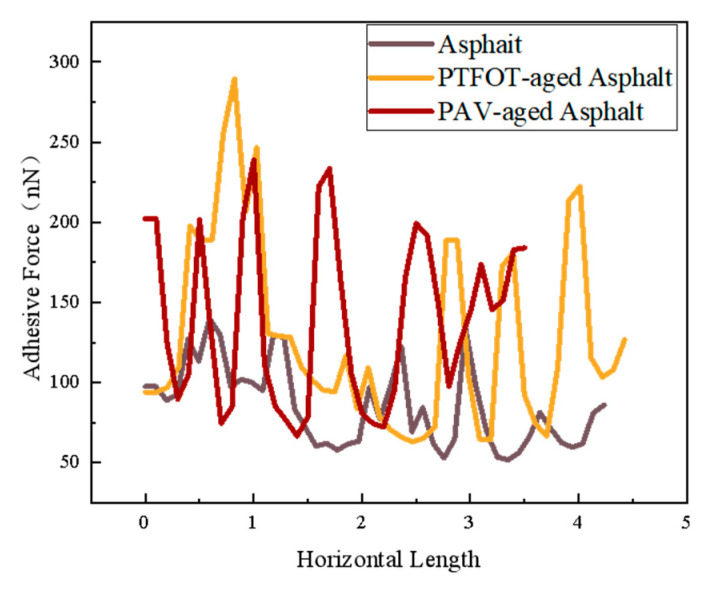
AFM adhesion distribution of SBS-modified asphalt with compatibilizer addition.

As shown in Figure 19, the smaller relative molecular weight of the continuous phase is prone to diffusion, which results in good adsorption with the probe, thereby improving the adhesion between it and the probe. The adhesion of the honeycomb structure portion of the asphalt with the addition of the compatibilizer was overall improved compared to the SBSMA. In the honeycomb structure, the adhesion of the honeycomb structure after RTFOT aging and PAV aging showed a wavy structure with peaks and valleys. In this case, the honeycomb structure adhesion of the asphalt in the original sample was greatly reduced [36].

After the addition of the compatibilizer, the AFM adhesion of the SBS-modified asphalt was more uniformly distributed (Figure 18), and the proportion of high adhesion areas within the honeycomb structure increased, indicating that the compatibilizer improved the molecular dispersion by inhibiting the π–π stacking (simulated decrease in the RDF peak, Figure 13). In addition, the decrease in adhesion after PAV aging was reduced (Figure 19), which is consistent with the conclusion of the simulation that the compatibilizer stabilizes the colloidal structure and delays aging and verifies its role in enhancing durability.

The experimental results above show the following:

(1) Solubility parameter: The trend of SPD measured in the experiment is consistent with the difference of the solubility parameter calculated in the simulation (Table 3), which confirms that the compatibilizer enhances the compatibility by adjusting the polarity of the components.

(2) Low-temperature performance: The decrease in the S value and the increase in the m value in the BBR experiment are directly related to the decrease in the diffusion coefficient (restricted molecular movement) and the elevation in CED (enhanced intermolecular interaction) in the simulation.

(3) Microstructure: The AFM adhesion distribution and simulated RDF analysis both indicated that the compatibilizer optimized the asphalt microhomogeneity by inhibiting gum aggregation.

In summary, the experimental data and the molecular dynamics simulation results were in high agreement at multiple scales, verifying the reliability of the model and the mechanism of action of the compatibilizer.

## 5. Conclusions

In this study, molecular dynamics methods were used to create molecular models of SBSMA or SBSMA was added to a self-developed compatibilizer. The compatibility and diffusion mechanism of the miscible process of SBS and the asphalt system were examined, and the thermodynamic and self-diffusion properties of various types of asphalt were compared. This study draws the following conclusions from molecular dynamics simulations:

(1) Molecular dynamics simulations successfully revealed the interfacial compatibility mechanism between SBS modifiers and asphalt at the molecular scale. The developed model demonstrated high fidelity to experimental observations, particularly in predicting cohesive energy density and diffusion coefficients, and the molecular structure model of the SBSMA built in this research is reasonable and effective, which can reflect the real state of the actual substance.

(2) When comparing the SBSMA and SBSMA systems, a compatibilizer was added, which resulted in a decrease in system instability, a reduction in intermolecular irregular motion in the blended system, and an increase in the cohesive energy density in the asphalt system. It reduced the intermolecular irregular motion of the blended system and lowered the system instability.

(3) The compatibilizer was added to asphalt to lower its free volume fraction and enhance intermolecular contact, which affects molecular motion and lowers the diffusion coefficient.

(4) The compatibilizer in the aromatic and saturated fractions of the combined effect of the dispersion of asphaltene hindered the ability of asphaltene aggregation, and, concurrently, molecular interactions with aromatic or saturated hydrocarbons (and saturated hydrocarbons with good mutual solubility) are better, resulting in the dispersion of aromatic and saturated hydrocarbons in the molecular mixture through asphaltene gelatin molecules. This enhanced the structural homogeneity of the asphalt binder, resulting in the formation of a more stable colloidal system, improved low-temperature cracking resistance, and aging resistance and durability.

(5) Experimental validation (solubility parameters, BBR low-temperature performance, AFM adhesion distribution) and molecular dynamics simulation results (CED, diffusion coefficients, RDF) agree on multiple scales, confirming the accuracy of the simulation predictions and providing a reliable framework for cross-scale studies of compatibilizer molecular design.

(6) The use of model simplifications with time scale limitations and force field and system size constraints may affect the accuracy of asphaltene aggregation behavior. Future combinations of all-atom models with extended simulation times, the development of a multiscale simulation framework, and experimental validation will optimize force-field parameters and expand the system size to reduce boundary effects.

## Figures and Tables

**Table 1:** Molecular structure characteristics of asphalt binder components.

SARA Component	Molecule Name	Number of Molecules	Molecular Formula	Molecular Weight (g/mol)	Mass Fraction (%)
Saturated fraction	Squalane	4	C_30_H_62_	422.9	5.2
	Hopane	4	C_35_H_62_	482.8	5.8
Aroma	PHPN	11	C_35_H_44_	464.8	15.7
	DOCHN	13	C_30_H_46_	406.8	16.2
Resin	Pyridinohopane	4	C_36_H_57_N	530.9	6.2
	Thio-isorenieratane	4	C_40_H_60_S	572.9	7.0
	Trimethylbenzene-oxane	5	C_29_H_50_O	414.7	6.4
	Quinolinohopane	4	C_40_H_59_N	554.0	6.8
	Benzobisbenzothiophene	15	C_18_H_10_S_2_	290.4	13.4
Asphaltene	Phenol	3	C_42_H_54_O	575	5.3
	Pyrrole	2	C_66_H_81_N	888.5	5.5
	Thiophene	3	C_51_H_62_S	707.2	6.5

**Table 2:** Technical specifications of compatibilizer.

Item		Unit	Test Value
Color		-	dark colored
Density at 15 °C		g/cm^3^	0.874
Flash point		°C	212.1
Chemical composition	Asphaltene	%	0.73
	Gum	%	9.53
	Aromatic	%	28.43
	Saturated	%	61.31

**Table 3:** Solubility parameters of SBS and different asphalt binder systems.

	SBS	Base Asphalt Binder	SBSMA System
Solubility parameters ((J/cm^3^)^1/2^)	18.088	19.024	17.872
SPD	-	0.936	−0.216

## Data Availability

The original contributions presented in this study are included in the article. Further inquiries can be directed to the corresponding authors.

## Abbreviations

SBS	Styrene–butadiene–styrene
SBSMA	Styrene–butadiene–styrene-modified asphalt
PS	Polystyrene
PB	Polybutadiene
Tg	Glass transition temperature
MD	Molecular dynamics simulation
MS	Materials Studio
SARA	Saturated, aromatic, resin, and asphaltene
NVT	Number of system atoms, volume, and temperature
NPT	Constant molecular number, external pressure, and temperature
CG	Coarse-graining
PBC	Periodic boundary conditions
RDF	Radial distribution function
DFT	Density-functional theory
CED	Cohesive energy density
SPD	Solubility parameter difference
MSD	Mean square displacement
